# An Audio Personal Health Library of Clinic Visit Recordings for Patients and Their Caregivers (HealthPAL): User-Centered Design Approach

**DOI:** 10.2196/25512

**Published:** 2021-10-22

**Authors:** Paul J Barr, William Haslett, Michelle D Dannenberg, Lisa Oh, Glyn Elwyn, Saeed Hassanpour, Kyra L Bonasia, James C Finora, Jesse A Schoonmaker, W Moraa Onsando, James Ryan, Martha L Bruce, Amar K Das, Roger Arend, Sheryl Piper, Craig H Ganoe

**Affiliations:** 1 The Dartmouth Institute for Health Policy & Clinical Practice Geisel School of Medicine Dartmouth College Lebanon, NH United States; 2 The Center for Technology and Behavioral Health Geisel School of Medicine Dartmouth College Lebanon, NH United States; 3 Department of Computer Science Dartmouth College Hanover, NH United States; 4 Department of Biomedical Data Science Dartmouth College Hanover, NH United States; 5 Department of Epidemiology Dartmouth College Hanover, NH United States; 6 Geisel School of Medicine Dartmouth College Hanover, NH United States; 7 Ryan Family Practice Ludington, MI United States; 8 Department of Psychiatry Geisel School of Medicine Dartmouth College Hanover, NH United States; 9 Patient Partner Lebanon, NH United States

**Keywords:** patient-centered care, health communication, audiovisual aids, user-centered design, software, natural language processing, patients, caregivers

## Abstract

**Background:**

Providing digital recordings of clinic visits to patients has emerged as a strategy to promote patient and family engagement in care. With advances in natural language processing, an opportunity exists to maximize the value of visit recordings for patients by automatically tagging key visit information (eg, medications, tests, and imaging) and linkages to trustworthy web-based resources curated in an audio-based personal health library.

**Objective:**

This study aims to report on the user-centered development of HealthPAL, an audio personal health library.

**Methods:**

Our user-centered design and usability evaluation approach incorporated iterative rounds of video-recorded sessions from 2016 to 2019. We recruited participants from a range of community settings to represent older patient and caregiver perspectives. In the first round, we used paper prototypes and focused on feature envisionment. We moved to low-fidelity and high-fidelity versions of the HealthPAL in later rounds, which focused on functionality and use; all sessions included a debriefing interview. Participants listened to a deidentified, standardized primary care visit recording before completing a series of tasks (eg, finding where a medication was discussed in the recording). In the final round, we recorded the patients’ primary care clinic visits for use in the session. Findings from each round informed the agile software development process. Task completion and critical incidents were recorded in each round, and the System Usability Scale was completed by participants using the digital prototype in later rounds.

**Results:**

We completed 5 rounds of usability sessions with 40 participants, of whom 25 (63%) were women with a median age of 68 years (range 23-89). Feedback from sessions resulted in color-coding and highlighting of information tags, a more prominent play button, clearer structure to move between one’s own recordings and others’ recordings, the ability to filter recording content by the topic discussed and descriptions, 10-second forward and rewind controls, and a help link and search bar. Perceived usability increased over the rounds, with a median System Usability Scale of 78.2 (range 20-100) in the final round. Participants were overwhelmingly positive about the concept of accessing a curated audio recording of a clinic visit. Some participants reported concerns about privacy and the computer-based skills necessary to access recordings.

**Conclusions:**

To our knowledge, HealthPAL is the first patient-centered app designed to allow patients and their caregivers to access easy-to-navigate recordings of clinic visits, with key concepts tagged and hyperlinks to further information provided. The HealthPAL user interface has been rigorously co-designed with older adult patients and their caregivers and is now ready for further field testing. The successful development and use of HealthPAL may help improve the ability of patients to manage their own care, especially older adult patients who have to navigate complex treatment plans.

## Introduction

### Background

Higher recall of medical information is associated with improved disease management, treatment adherence, and higher patient satisfaction [1,2]. Recall, however, is often low, with 40%-80% of medical information from a clinical visit being forgotten immediately by patients [3-8]. Although the poor recall of medical information is pervasive, it is most acute among older adults. As people age, they process information more slowly and have reduced working memory [9,10]. Older patients experience more challenges in recalling drug information, treatment recommendations, appointments, and disease information [11], especially those with multimorbidity [1,6,8,12-14] who report the *endless struggle* of managing their conditions [15,16]. Poor recall also impacts caregivers [17]. In a recent national survey of caregivers in the United States, 84% of respondents wanted more information on caregiving topics [17].

The last decade has seen significant efforts to increase patient access to medical information, especially clinic visit information. Mandated initially by the Health Information Technology for Economic and Clinical Health Act’s meaningful use standards, clinics across the United States now offer patients an after-visit summary (AVS) [18]. The AVS is a summary of the clinic visit generated from the electronic medical record, printed during visits, or available via the patient portal and includes diagnoses, medications, allergies, clinician visited, and clinician comments. Although intended as a means of promoting self-management, there have been concerns about the AVS, including accuracy of medication lists, layout, and use of medical terminology on the AVS [19]. This is particularly challenging for patients who often report low health literacy and struggle with exclusively text-based information [2,3,20-22]. AVS can also represent a significant burden on clinician workload [7,23]. These factors have resulted in low AVS use [24]. An adjunct to the AVS may exist in recordings of the clinic visit.

Patients rely on verbal communication with their doctor [8] and some are now audio recording clinic visits to capture this valuable information [25-27]. In response, a small but growing number of clinics across the United States are beginning to offer patients recordings of clinic visits. Systematic reviews found that access to recordings leads to increased patient and family engagement, understanding, and recalling visit information; reduced anxiety; increased satisfaction; improved treatment adherence; reduced patients’ clinic phone calls; and reduced decisional regret [25,28-33].

The absence of a safe and secure recording system is a barrier to the broader uptake of clinic recordings [27]. A recording provides all the visit details, yet navigating recordings is a challenge, as the benefit “depends on picking out...the crucial points...” of the visit [27]. Unstructured visit information increases the risk of overwhelming patients [27,34].

Electronic personal health libraries (PHLs) may be a solution, as they allow patients to manage, maintain, and organize health information on the web [34,35]. PHLs can range from medical records that patients can access tethered to a health system to stand-alone platforms where patients collect and manage their own data. PHLs are becoming more advanced through the application of data science methods such as natural language processing (NLP) [36]. These methods can identify patterns in unstructured data and classify text based on its meaning. Such NLP methods have been used to predict hospital readmissions [37], future radiology utilization [38], and medical conditions in clinical decision support systems [39]. In PHLs, data science methods have been used to automatically provide tailored information via guided searches for disease and self-care information [36]. Despite the availability of these methods, existing PHLs are yet to facilitate the integration of clinic recordings [40].

To address this gap, we planned to develop an audio PHL to facilitate the acquisition, organization, and management of clinic audio recordings—HealthPAL (personal audio library). On the basis of a review of patients’ information-seeking behavior and needs [41], the basic features of HealthPAL include (1) identifying, organizing, and tagging elements of the clinic visit audio recording deemed important to patients; for example, parts of the visit recording where medications are mentioned would be automatically highlighted for patients; (2) a search function, allowing end users to search for information from their visit; and (3) linkage of key medical terms from the clinic visit audio recordings to trustworthy, layperson resources such as MEDLINE Plus, which can be retrieved, organized, edited, and shared by patients. For example, a hyperlink to learn more about the medication mentioned would be available to the patient. In the system’s background, a transcript of the medical visit is automatically generated using speech-to-text software. However, because of concerns of inaccurate speech-to-text potentially providing incorrect written medical information, we chose not to expose full transcript text in our design.

Our user-centered design work falls in the history of design studies around *meeting browsers* [42]—software multimedia browsers of meeting recordings and associated meeting artifacts—where HealthPAL is the first to consider design and usability for the specialized context of patients meeting with their primary care provider. *Classroom 2000* [43] initiated the modern genre of meeting browsers, which focused on capturing a *recording* of a meeting or classroom lecture and its context, often focusing on live audio and video and linking to slides of a PowerPoint-like presentation, with some allowing users to add their own annotations or notes. Early evaluation work with a meeting browser found that such a system can allow users to more accurately answer questions about a meeting [44], whereas features that allow the user to focus on key phrases allowed them to answer questions about the audio content more quickly [45]. We hypothesize similar uses of annotated audio recordings in health care.

### Objective

This study reports on the development of the HealthPAL user interface (UI). Results from our data science models are reported elsewhere. By adopting a user-centered design framework, we engaged end users throughout the iterative development of HealthPAL [40,41]. We followed the usability specification and evaluation framework developed by Yen and Bakken [46], which consisting of 5 stages: (1) system requirements, (2) system component development, (3) usability evaluation in controlled settings, (4) pilot field testing, and (5) large-scale field testing. We report on steps 1-3 in this paper. We hypothesized that through user-centered design, HealthPAL would be highly usable with high end user satisfaction.

## Methods

### Study Design: Overview

**Figure 1:**
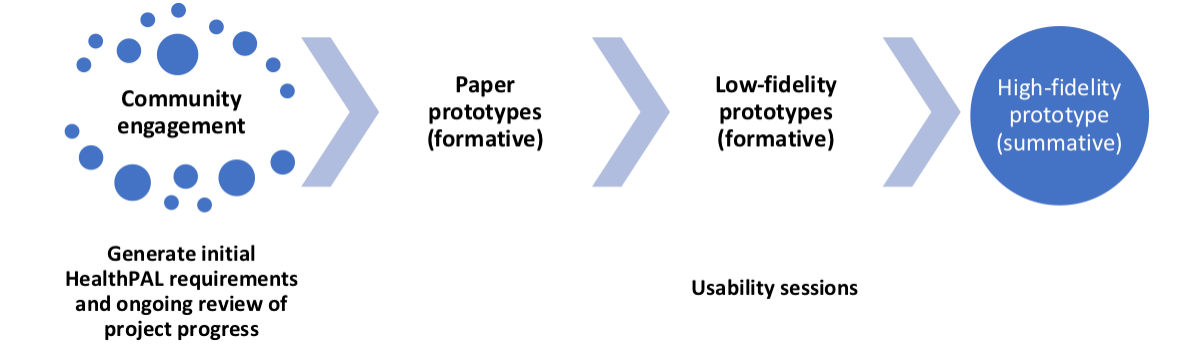
Overview of the user-centered design stages of HealthPAL.

Our approach incorporated UI development conducted through 5 iterative rounds of *usability sessions*. During the sessions, participants were asked to play the role of a patient or caregiver and complete a series of typical tasks within HealthPAL. The UI was iteratively refined in each round, with additional tasks added to assess the newly added features. We began with the paper prototype and formative sessions (rounds 1-3) in which participants worked with paper and low-fidelity software prototype designs before moving to the summative software sessions (rounds 4 and 5) in which they interacted with higher fidelity prototypes of the software (Figure 1). The sessions were structured so that participants would first listen to a fictitious clinic visit recording, and in the final summative software session (round 5), patients used their actual clinic visit recordings. Participants were presented with a set of typical user tasks to complete in the system being tested during their session.

Before each new iterative round of user testing, the research team completed heuristic evaluations and cognitive walkthroughs with HealthPAL to mitigate common usability problems before working with participants.

### Settings

Participants were recruited from the Upper Valley of New Hampshire and Vermont between 2016 and 2019. Individuals were recruited from multiple settings, including public libraries, the Dartmouth-Hitchcock Medical Center (DHMC) simulation and human-computer interaction lab, Dartmouth-Hitchcock (D-H) Heater Road Primary Care, D-H Patient and Family Advisors group, the D-H Aging Resource Center, and a local senior living community. This study was approved by the Committee for the Protection of Human Subjects, Dartmouth College and the D-H Health Human Research Protection Program (Committee for the Protection of Human Subjects Study #30397, 30531; D-HH HRPP 00030531).

### Participants

Participants included individuals who represented the views of patients and caregivers. All participants were 18 years or older, able to communicate in English, and able to provide informed consent. Individuals with serious mental illness, self-reported significant uncorrectable hearing or visual impairments, or significant cognitive impairment (score of 4 or less on a 6-item screener) [47] were excluded from the study. Caregiver participants were individuals who self-identified as having previously cared for a family member or loved one. In the final round of user testing, we also recruited primary care clinicians to record the clinic visits of patients to be used during the usability evaluation sessions. Consented clinicians identified patients who met the eligibility criteria (18 years or older with ≥2 chronic health conditions) and who were facing a treatment decision or were discussing a diagnosis or medication; patients visiting solely for a procedure, such as blood draw, were excluded.

Our project initially focused on the general public, but additional funding received from the National Library of Medicine during the study allowed us to expand the proposed features and focus on older adults who account for the greatest use of health care and may benefit the most from the proposed system. Therefore, we oversampled older adults (≥65 years) and focused on this group in later rounds. As we moved to later rounds of user testing, it was also necessary for participants to have internet access at home to review the recording system before the usability session. A US $25-$30 honorarium was provided to participants. We targeted 5 individuals per round, a sample size that is considered adequate to detect up to 80% of usability issues [48,49]. All participants provided written informed consent. Participants from previous rounds could not participate in later rounds to reduce the potential impact of learning effects inflating usability evaluations.

### Recruitment

In the initial paper prototype and formative sessions (rounds 1 and 2), we recruited participants from the DHMC Patient and Family Advisors group and from public spaces at a local library. Participants were approached by a research team member; informed about the project; and if interested, they were taken to a private space, they provided consent, and they began the usability session. For the final paper prototype and formative session (round 3), we focused on older adults, and recruitment moved to the Aging Resource Center and an older adult living community. Participants were sent physical mail and an email to inform them about the project. The screening was conducted by telephone with interested participants to determine eligibility, and participants were met on the day of the session to complete informed consent before beginning the usability session.

In the summative software sessions (rounds 4 and 5), we specifically targeted individuals from both *patient* and *caregiver* stakeholder groups separately. Caregivers were recruited from the Aging Resource Center using the procedures described above. Patients were recruited from a local primary care clinic, identified by participating clinicians, and received a screening telephone call from a research team member. Eligible patients were asked to come to the clinic 30 minutes earlier than their appointment to complete the informed consent process; once they provided consent, their clinic visits were recorded using the software. Participants were then asked to meet with the research team within a week to complete the usability session.

### Community Engagement

In addition to the 5 rounds of user testing, 2 *Lunch and Listen* workshops were conducted with patients and family volunteers from DHMC, Lebanon, New Hampshire. These workshops were codeveloped and led by our study team’s patient partners (RA and SP) to discuss key system concepts with community members. Responses from these workshops informed the initial layout, features, and desired functionality of the HealthPAL system that was tested during the usability sessions.

### Usability Sessions

#### Overview

All sessions began with a description of the usability session, and participants were asked to *think aloud* [50,51] as they completed the tasks with the software. Paper prototype sessions were video-recorded along with participant and facilitator audio, and in later rounds where software prototypes were used by participants, screen video and audio were recorded to capture participants’ use of the prototype and their verbal feedback. Upon completion of the round-specific tasks, participants completed a semistructured interview about the system and desired functionality. The session facilitator in the room made written field notes related to participants’ interactions with the prototype.

#### UI Design

In all sessions, the UI primarily consisted of 2 pages. The first page was an interface allowing the user to choose which recordings the user is viewing (eg, choosing between their own visit recordings and the visit recordings of someone who they act as a caregiver for). The second page offered basic playback features (eg, play and pause, rewind, skip forward or back 10 seconds, and mute) for a visit recording. In addition, on that page, individual audio segments of the visit recording were *tagged* for 8 classes of information: diagnosis, follow-up, medication, patient education, recommendation, signs, symptoms and problems, test and imaging, and treatment options. The user could click on a segment to start playback at that point. New features were added to each round based on the user feedback.

#### Fictitious Clinic Visit Recordings

Fictitious primary care visit recordings were created and used throughout the user testing sessions, except in the final round of patient sessions. We created 2 fictitious characters, Chris Hill, a 58-year-old male patient, and his 81-year-old mother Linda, both of whom met with a fictitious primary care clinician named Dr Adams. The fictitious clinic visit recordings for both the characters were produced by rerecording 2 real primary care clinic visits, stripped of patient identifiers (a description of the recording is provided in Textbox 1).

**Fictitious clinic recordings.**

**Chris Hill**

- Chris’s recording was a clinic visit of approximately 7 minutes in which Chris and Dr Adams discuss Chris’s allergies, and Dr Adams suggests increasing his dosage of Flonase. They also talk about Chris’s stomach pain, and Dr Adams suggests increasing his dosage of Omeprazole. Chris talks about his emergency room visit from when he was having bad stomach pain, and Dr Adams reviews some test results from that visit.

**Linda Hill**

- Linda’s recording was a clinic visit of approximately 11 minutes in which Linda and Dr Adams discuss the pain in her foot and back. Linda tells Dr Adams that she has stopped taking the nerve pain controller Gabapentin because of the side effects, but Dr Adams suggests Linda to try it again. They also discuss Linda’s arthritis and the ganglion cyst on her hand. Dr Adams informs her that the cysts can be surgically removed if they bother her and also suggests that she visits a pain clinic for overall pain control.

#### Paper Prototyping and Formative Usability Sessions (Rounds 1-3)

##### Overview

The initial round began with a paper prototype before moving onto the low-fidelity prototypes of the software in rounds 2 and 3. During the paper prototype sessions, the facilitator adopted a *Wizard of Oz* technique, where they played back the appropriate recorded patient clinic visit audio in response to the participant’s interactions with the prototypes [52]. Participants in these sessions (rounds 1-3) were asked to complete tasks in both patient and caregiver roles.

##### Patient Role

Participants were given the role of Chris. They began the round by listening to the entire fictitious audio recording for Chris before being presented with the paper prototype and before being asked to complete the tasks (Textbox 2). This was designed to replicate the experience of a patient who was present during the visit.

**Example of a role-based scenario and task for participants to complete in the prototypes.**

**Role**

- In this study, you will play the role of *Chris*. Chris Hill is a 58-year-old male patient of Dr Adams, who last saw this doctor on June 20, 2017—1 week ago. Chris’s mother, Linda, who is 81 years old, also sees Dr Adams.

**Scenario**

- Chris’s doctor creates audio recordings of patients’ visits and provides a web-based software app for patients to listen to their own visit recordings after they leave from each visit. Patients can also grant permission for other family members or caregivers to listen to their visit recordings. Chris also has access to listen to his mother’s clinic visits, so that he can stay up to date on her health. Chris knows that his mother visited Dr Adams on June 14, 2017—her most recent visit. Chris also knows his mother and Dr Adams discussed possible surgery for the pain she has in her hand. They also discussed her restarting a medication for her foot pain that Linda had previously stopped taking. Later you will be asked to find and listen to important audio segments of her most recent visit in the website prototype.

**Task**

- Find where Dr Adams talks about considering surgery for Linda’s hand pain (ganglion cyst) in her most recent visit. When you believe you are finished with the task, say “I found it” aloud.

##### Caregiver Role

Once participants completed tasks with Chris’s recording, they were asked to find Linda’s recordings in the system and asked to complete a series of similar tasks. They did not listen to Linda’s recording before the tasks, replicating the experience of a caregiver who was not present at a visit.

#### Summative Software Usability Sessions (Rounds 4 and 5)

Starting from round 4, we recruited individuals who identified as either caregivers or patients and asked them to play only that role. All participants completed the same tasks, which required finding and listening to important parts of Linda’s recording and new features, including finding and using hyperlinks to additional information on a medical concept. In the final round of user testing, round 5, patients reviewed a recording of their primary care visit with a high-fidelity version of HealthPAL with all desired features; caregivers continued to review fictitious recordings. In parallel with UI development, we are developing NLP models to automatically annotate the classes of information in recordings. However, at this stage of development, we used human transcription and manual tagging of recordings by 2 clinically trained annotators (JAS and WMO). Patients were emailed a link to log in to the system to access their recording within 3 days of their appointment. Participants were then asked to return for an in-person session to demonstrate how they used the system and complete a set of specific tasks within the system.

### Data Collection and Analysis

#### Overview

We collected data on participant age, sex, race, ethnicity, and educational attainment for all rounds of usability testing. The System Usability Scale (SUS) [53] was administered from round 3 onward, as we moved to a web-based version of the software. Usability evaluation metrics included the SUS, critical incidents [54], and task completion ratios. We also gathered data on participant suggestions and views—general attitudes participants had toward the system.

#### Task Completion

For each task given to participants, we recorded whether the participant completed the task on their own, completed the task with help from the facilitator, or did not complete the task.

#### Critical Incidents

Recordings were coded for key critical incidents related to usability and interface design. These incidents included whenever a participant verbally or implicitly made known that they were struggling with an action, whether it was controlling the audio or finding the correct segment, regardless of task completion. Critical incidents also included whenever a participant took an action that deviated from the expected path, such as when participants clicked on a hyperlink instead of playing an audio segment.

#### Participant Suggestions

We recorded any feedback that participants provided about improving the website. This feedback included suggestions such as how the participants would like a feature to work or what the participants were expecting to see on a certain page.

#### Participant Views

We recorded any comments that participants made regarding their general attitudes toward the system. These comments included positive remarks, such as how the system could be beneficial for keeping track of important health information, and concerns, such as the security and privacy of their health information.

Descriptive statistics of median and range were used for continuous data, and proportions and ranges were used for categorical data. SUS scores were calculated on a scale of 0-100, with a score of >68 indicating above-average satisfaction with the usability of a system [55-57]. Descriptive summary statistics of the above-mentioned usability metrics were guided by the TURF (Task, User, Representation, and Function) framework [58], with a focus on task completion and system usability. We used summative content analysis to identify key issues and suggestions from the usability sessions. Transcripts from usability sessions were reviewed by 2 coders (LO and CHG). Commonly identified suggestions, views, and critical incidents were coded and grouped into the main themes.

### Software Architecture

**Figure 2:**
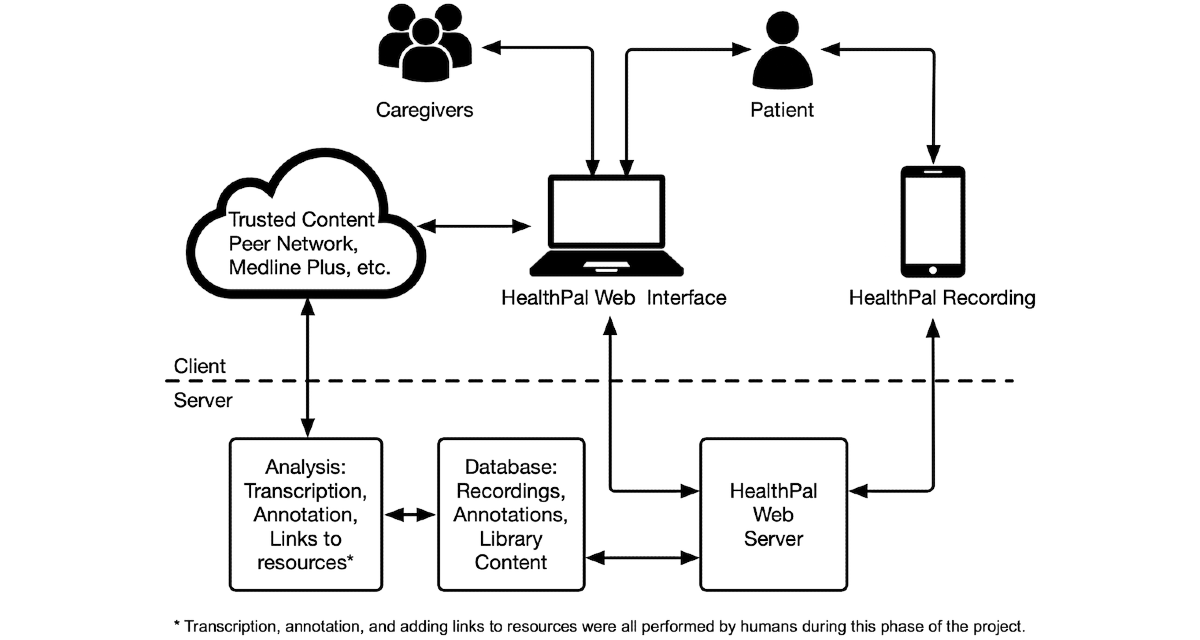
HealthPAL system architecture.

We built the software prototype for this work as a web app, using the mature and widely adopted Ruby on Rails app development framework. The app is hosted on Dartmouth’s secure server infrastructure, and all client-server communications are encrypted using an HTTPS protocol. In addition, the app software communicates with a PostgreSQL relational database, hosted on Dartmouth’s infrastructure, and app data were encrypted at rest. Using this architecture, we were able to securely deliver the app to a range of devices, supporting both audio recording and playback while avoiding storing audio or other protected health information on users’ devices. This architecture also allowed us to rapidly implement design changes that were made based on the results of each round of user testing (Figure 2). By using the Git version control system and an automated app deployment pipeline, we were able to track every deployment of the app code, thus producing a history of precisely which version of the software was running at any given time. The app prototype is available as open-source software under the MIT license. The source code can be obtained at the website [59].

## Results

### Participant Characteristics

**Figure 3:**
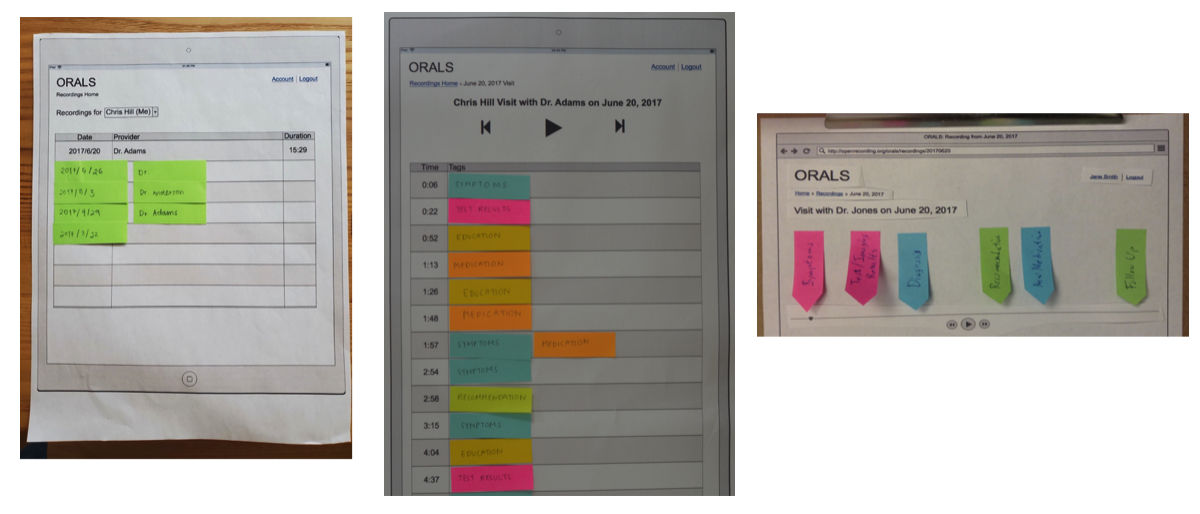
Initial paper prototypes of HealthPAL (round 1)—interface for finding a patient’s recording (left) and playing back the recording (center and right).

**Figure 4:**
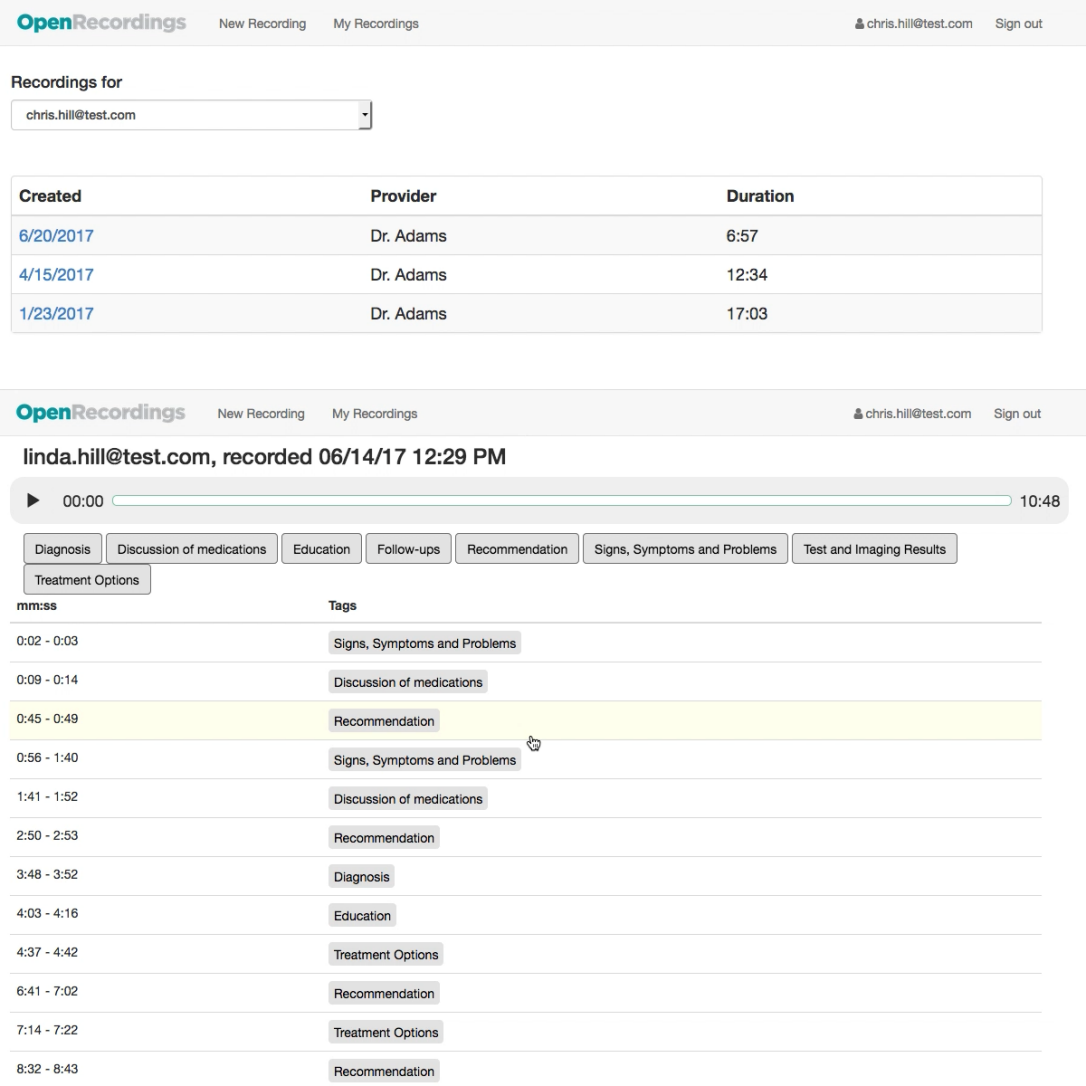
Initial software prototype (round 2)—interface for finding a patient’s recording (top) and playing back the recording (bottom).

**Figure 5:**
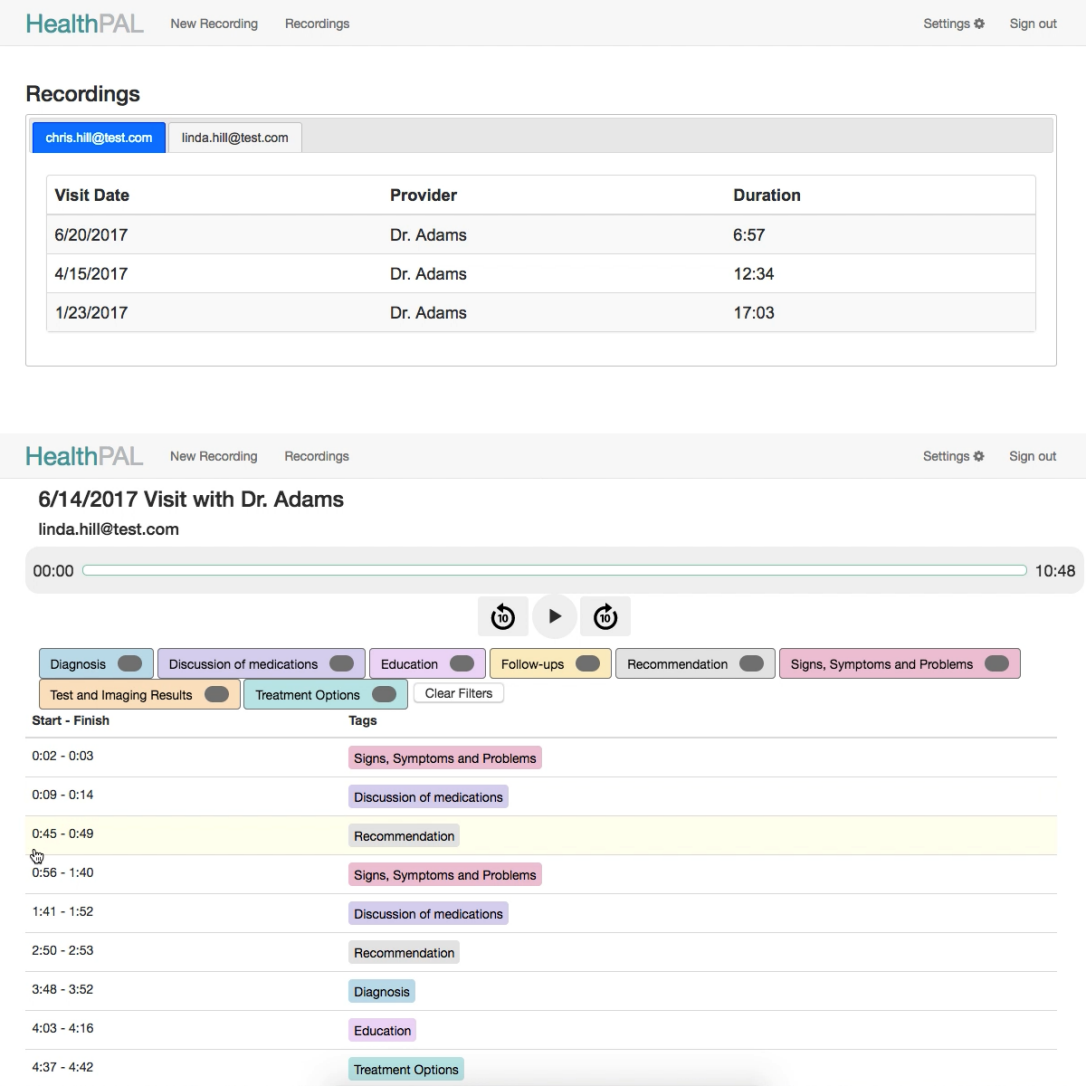
Updated software prototype (round 3)—interface for finding a patient’s recording (top) and playing back the recording (bottom).

**Figure 6:**
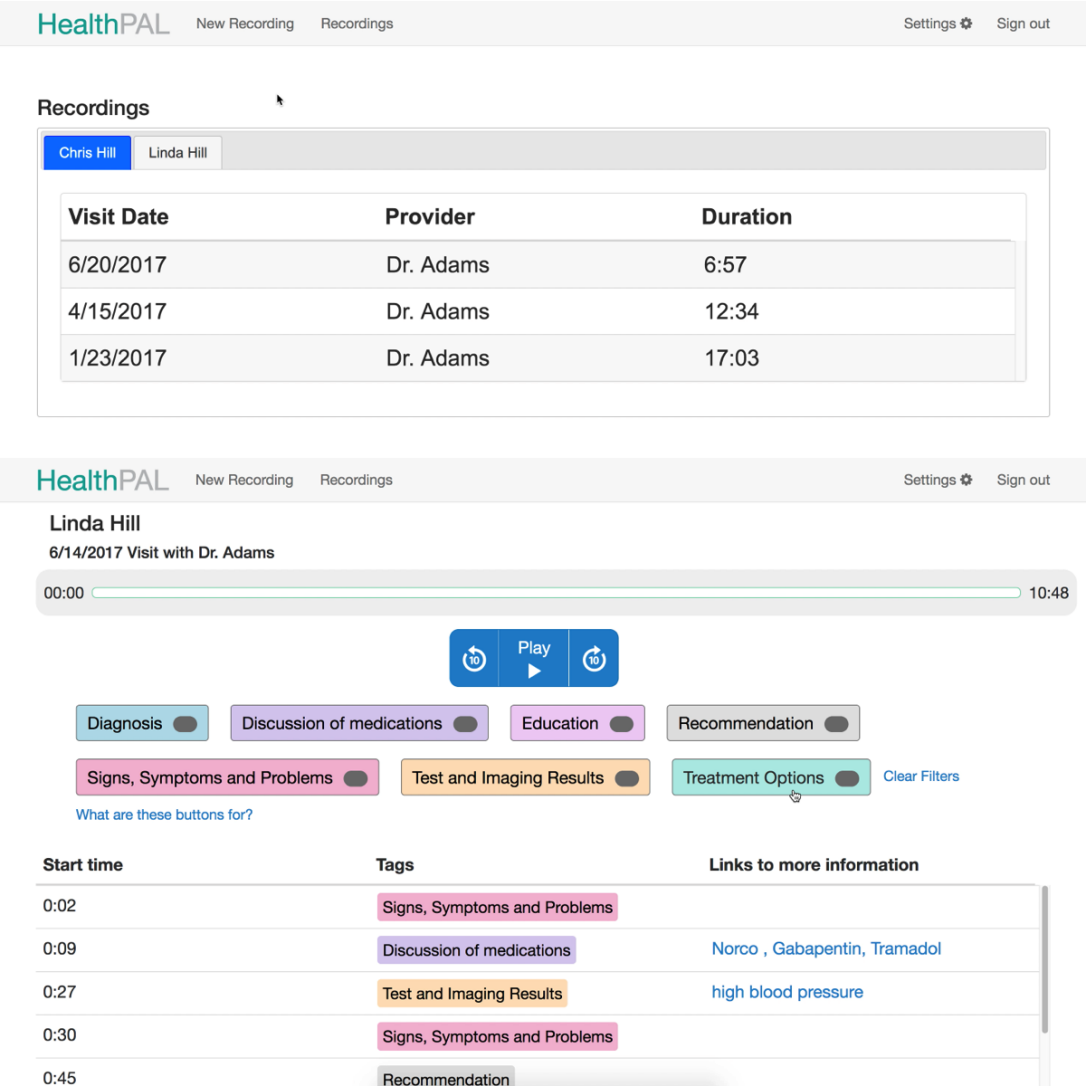
Updated software prototype (round 4)—interface for finding a patient’s recording (top) and playing back the recording (bottom).

**Figure 7:**
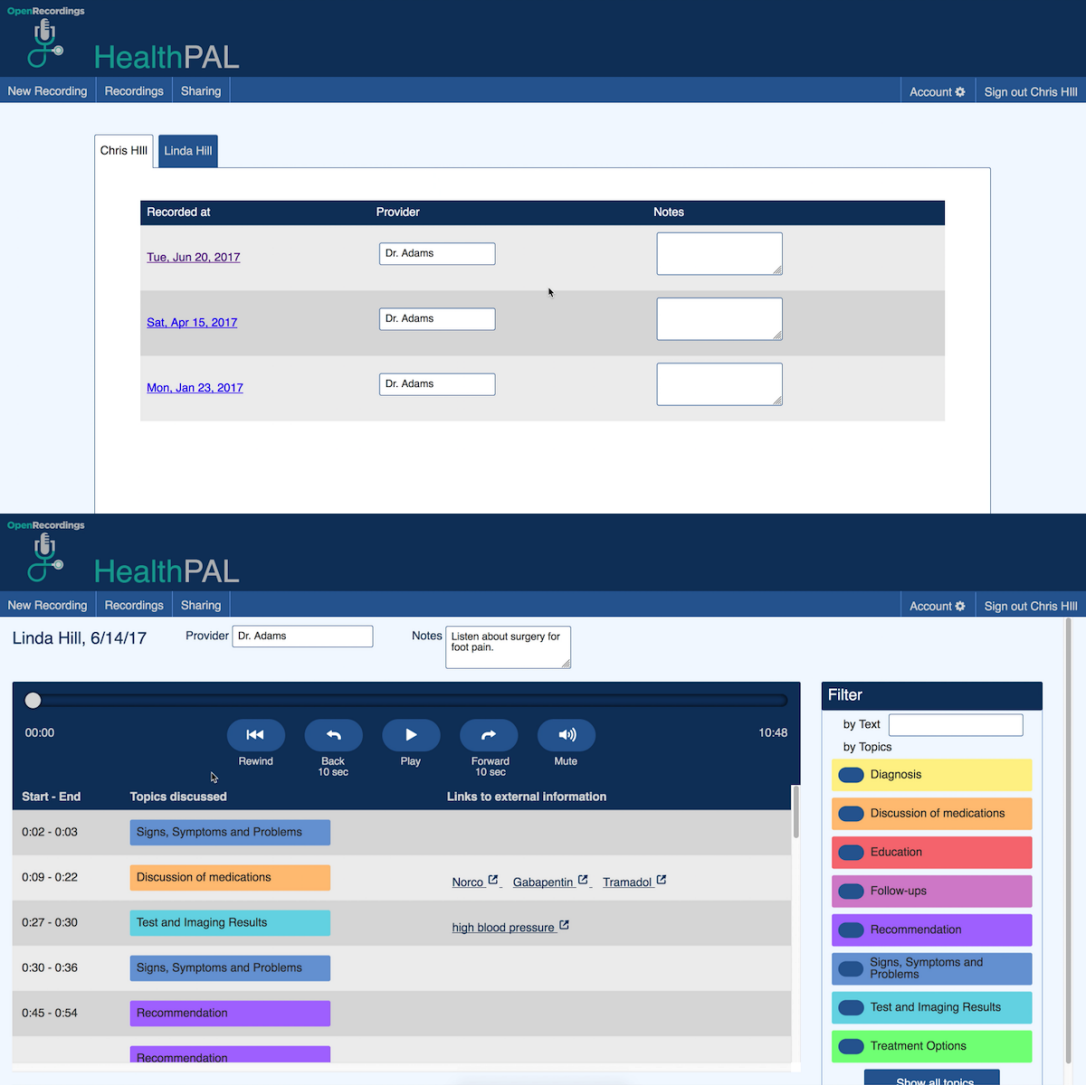
High-fidelity version of the software prototype (round 5)—interface for finding a patient’s recording (top) and playing back the recording (bottom).

We completed usability sessions with 40 participants (including 10 self-identified caregivers) from October 2017 to May 2019. Participants were predominantly White non-Hispanic (39/40, 98%), with a median age of 68 years (range 23-89 years). There were 63% (25/40) female participants, and most participants (37/40, 93%) had some college education or higher (Table 1). The evolution of the UI during rounds of user testing is shown in Figures 3-7.

**Table 1:** Participant demographics (N=40).

Participant characteristics		Round 1 (n=8)	Round 2 (n=6)	Round 3 (n=5)	Round 4			Round 5	
					CG^a^ (n=5)	P^b^ (n=6)	CG (n=5)		P (n=5)
Age (years), median (range)		54 (23-80)	48 (26-81)	80 (68-89)	70 (52-83)	71 (66-88)	72 (59-77)		62 (30-67)
Females, n (%)		3 (38)	3 (50)	3 (60)	5 (100)	3 (50)	4 (80)		4 (80)
**Race^c^, n (%)**									
	Hispanic White	0 (0)	0 (0)	1 (20)	0 (0)	0 (0)	0 (0)		0 (0)
	Non-Hispanic White	8 (100)	6 (100)	4 (80)	5 (100)	6 (100)	5 (100)		5 (100)
	Black or African American	0 (0)	0 (0)	0 (0)	0 (0)	0 (0)	0 (0)		1 (20)
**Education, n (%)**									
	High school or no degree	0 (0)	1 (17)	0 (0)	0 (0)	0 (0)	0 (0)		0 (0)
	High school graduate	1 (13)	1 (17)	0 (0)	0 (0)	0 (0)	0 (0)		0 (0)
	Some college	1 (13)	1 (17)	0 (0)	1 (20)	0 (0)	0 (0)		1 (20)
	College degree (bachelors or associate)	2 (25)	2 (33)	3 (60)	3 (60)	2 (33)	2 (40)		4 (80)
	Masters, doctorate, or professional school	4 (50)	1 (17)	2 (40)	1 (20)	4 (67)	3 (60)		0 (0)

^a^CG: caregiver role.

^b^P: patient role.

^c^More than 1 response allowed.

### Evaluation Outcomes

#### Task Completion and System Usability

Task completion followed an inverted *U*-shaped distribution, with high completion rates in the early and later rounds and a drop in performance in between (Table 2). However, as new features were added, some tasks required further assistance (ie, find and play where medications are discussed on the recording), whereas the initial introduction of hyperlinks to further information resulted in poor task completion for these tasks. By round 5, the majority of tasks were completed without assistance. SUS assessments were introduced in round 3; the median SUS score improved across the rounds: round 3, 40 (range 38-68); round 4, 73 (range 35-100); and round 5, 78 (range 20-100). Scores for caregivers were lower than those for patients in both rounds 4 and 5.

**Table 2:** Task completion ratios and system usability (N=40).

Evaluation outcomes			R^a^1 (n=8)		R2 (n=6)		R3 (n=5)		R4 (n=11)					R5 (n=10)		
									CG^b^ (n=5)		P^c^ (n=6)		CG (n=5)			P (n=5)
**Task (completed task alone:completed task with help:did not complete task)**																
	Find visit recording task	4:3:1		3:2:1		2:3:0		3:2:0		5:1:0		4:1:0			4:1:0	
	Find or play health issue task	8:0:0		5:1:0		3:1:1		2:2:1		4:2:0		2:3:0			4:1:0	
	Find or play medication task	8:0:0		5:1:0		0:3:2		1:4:0		6:0:0		5:0:0			4:0:1	
	Find more about health issue task	N/A^d^		N/A		N/A		2:1:2		1:0:5		5:0:0			3:0:2	
	Find more about medication task	N/A		N/A		N/A		3:1:1		3:0:3		5:0:0			5:0:0	
**Task completion, n (%)**																
	Total completed task (alone or with help)	23 (96)		17 (94)		12 (80)		21 (84)		22 (73)		25 (100)			22 (88)	
	Completed task with help	3 (13)		4 (22)		7 (47)		10 (40)		3 (10)		4 (16)			2 (8)	
SUS^e^ score (0-100), median (range)			N/A		N/A		40^f^ (38-68)		73 (35-100)		75 (55-100)		65 (20-90)			95 (43-100)

^a^R: round.

^b^CG: caregiver role.

^c^P: patient role.

^d^N/A: not applicable.

^e^SUS: System Usability Scale; not administered in the first 2 rounds.

^f^Missing data for 1 person.

#### Critical Incidents

A summary of critical incidents (challenges that prevented participants from completing a task independently) is presented in Table 3. The greatest number of challenges was observed in round 4, where hyperlinks were added. This resulted in confusion in finding sections of audio recordings to play using tags and using associated hyperlinks to find further information. When asked to find additional information about a medical term in the visit recording (with our intent being they use the hyperlink we provided in HealthPAL), some participants simply opened a new tab in the browser and conducted a web search; we counted these responses as not completing the task. Further refinements to the UI resulted in a lower proportion of critical incidents by round 5.

**Table 3:** Summary of key critical incidents that occurred during user testing (N=40).

Critical incidents^a^	R^b^1 (n=8), n (%)	R2 (n=6), n (%)	R3 (n=5), n (%)	R4 (n=11), n (%)			R5 (n=10), n (%)	
				CG^c^ (n=5)	P^d^ (n=6)	CG (n=5)		P (n=5)
Issues switching to or from another user’s recordings	6 (75)	3 (50)	4 (80)	1 (20)	—^e^	1 (20)		—
Issues understanding which user the recordings belonged to	3 (38)	2 (33)	4 (80)	2 (40)	1 (17)	1 (20)		—
Issues navigating through the site	2 (25)	1 (17)	0 (0)	4 (80)	2 (33)	1 (20)		1 (20)
Issues finding the appropriate segment	7 (88)	4 (67)	4 (80)	2 (40)	2 (33)	1 (20)		0 (0)
Issues controlling or traversing the audio	5 (63)	0 (0)	5 (100)	5 (100)	5 (83)	3 (60)		3 (60)
Issues using the filters by topic	—	3 (50)	2 (40)	4 (80)	2 (33)	2 (40)		2 (40)
Issues using hyperlinks to find additional information	—	—	—	5 (100)	4 (67)	3 (60)		2 (40)
Issues using the filters by text	—	—	—	—	—	3 (60)		1 (20)

^a^Proportion of sessions with a critical incident.

^b^R: round.

^c^CG: caregiver role.

^d^P: patient role.

**^e^**Feature not available or not part of testing.

#### Suggestions

Participants made several suggestions regarding improvements and functionality of the system (Table 4). These suggestions were grouped into common themes across the rounds and were used to improve the UI and functionality. By round 5, few additional suggestions emerged.

**Table 4:** Proportion of key suggestions given during user testing by category (N=40).

Suggestion	R^a^1 (n=8), n (%)	R2 (n=6), n (%)	R3 (n=5), n (%)	R4 (n=11), n (%)			R5 (n=10), n (%)	
				CG^b^ (n=5)	P^c^ (n=6)	CG (n=5)		P (n=5)
Suggestions for making segments easier to find within a recording	7 (88)	4 (67)	2 (40)	2 (40)	2 (33)	2 (40)		1 (20)
Suggestions for making specific visits easier to find	5 (63)	2 (33)	3 (60)	0 (0)	0 (0)	1 (20)		1 (20)
Suggestions to make switching between recordings of different users more intuitive (ie, switching from personal to loved one’s recording)	4 (50)	3 (50)	3 (60)	0 (0)	—^d^	1 (20)		—
Suggestions for supplementary text to include with recordings	3 (38)	3 (50)	2 (40)	3 (60)	0 (0)	1 (20)		2 (40)
Suggestions to make audio controls more intuitive	2 (25)	2 (33)	2 (40)	1 (20)	2 (33)	1 (20)		0 (0)
Suggestions to improve the filter by topic feature	—	1 (17)	0 (0)	1 (20)	0 (0)	3 (60)		0 (0)
Suggestions to improve the hyperlink feature	—	—	—	1 (20)	1 (17)	2 (40)		0 (0)
Suggestions to improve the filter by text feature	—	—	—	—	—	4 (80)		1 (20)
Suggestions for additional features	2 (25)	3 (50)	3 (60)	2 (40)	3 (50)	2 (40)		4 (80)

^a^R: round.

^b^CG: caregiver role.

^c^P: patient role.

^d^Feature not available or not part of testing.

#### Participant Views of an Audio PHL

Participants were overwhelmingly positive about the proposed system. Participants identified several benefits of having a visit recording. These included better recall of information for patients and the use of recordings as a historical artifact that could be revisited. For example, a participant said*,* “Because I had breast cancer 25 years ago and I’d like to go back and say, ‘What was that now? What did they say?’” [r3p05]. Communication of clinic visit information to caregivers was considered a significant benefit, as a caregiver whose mother has cognitive issues stated, “it’s cumbersome to try and get it from the doctor, so to have that in a place where you can go and access it [would be helpful]” [r4p04]. The added benefit of recording in comparison with written summaries was also mentioned. A participant considered written summaries as *minimal*, whereas another mentioned the ability “to listen to how the doctor said something and how much emphasis he or she was giving...” [r4p09] to clarify instructions; this reflects the added value recordings bring, including the information communicated through voice inflections. The use of hyperlinks was identified as an important feature as “you’re getting the information a whole lot quicker than going and sifting through what Google results come up” [r6p03].

Some concerns were also raised. These included the need to keep the UI as user-friendly as possible and not assume “computer capability” [r3p01]. Concerns of information privacy were also raised, “You made this easy for an outside user to access some elements of it – you wouldn’t want to let them in the whole thing” [r3p03].

Even when recordings would be shared with family members, patients reported the need for some caution: “I would want to be very careful about inviting relatives to get into the act” [r3p03]*.* It was mentioned that enabling features that allow partial sharing of recording may reduce this concern, which is not currently a function available in the system. Finally, there was concern from participants about the clinician giving permission for recording:

I can’t imagine him agreeing to it, my parents’ physician...I don’t know, it could be used, in an odd way, against the doctor.

r5p05

### Key Changes to HealthPAL Across Rounds

Textbox 3 outlines key changes made to the system and functionality added in response to user feedback, including color coding and highlighting of information tags, adding more prominent play and pause buttons, creating a clearer structure for switching between user accounts, adding tag filtering and descriptions, adding a 10-second forward and rewind control, a help link, and a search bar. Following our final round of user testing, we made minor modifications to clarify the elements of the interface that should be *clicked* for playback versus hyperlinks to new external information that was approved by our patient partners (RA and SP). Finally, a combination of user feedback and an attempt to optimize our approach to annotations, we reduced the information classes to medication, medical condition, test and imaging, and treatment and procedures.

**Summary of key changes to HealthPAL user interface.**

**Prototype Description and Updates**

**Figure 8:**
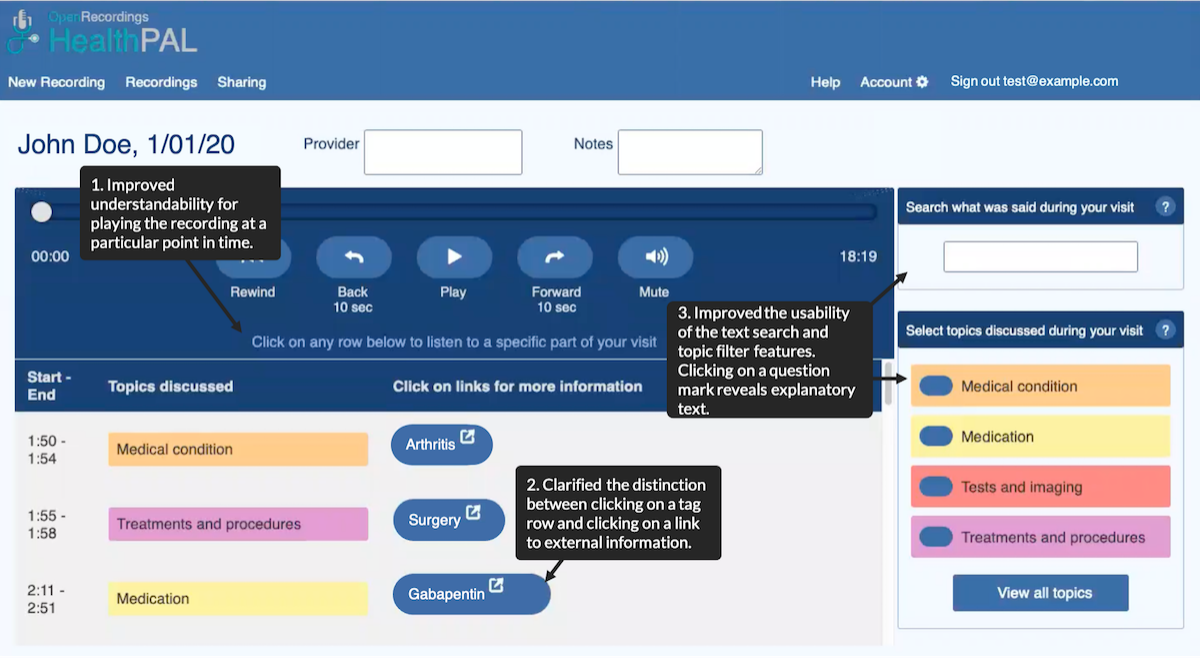
Final version of the software playback user interface.

- Round 1
  Initial paper prototype with audio controlled by the facilitator (Figure 3)
  User quotes
    “If there’s a way to get a finer level of detail [in the topic tags], that would probably be helpful.” [r1p03]
    “[I would like] some sort of indication of where I am in the recording.” [r1p05]
  Round 2
    Initial software prototype based on feedback from round 1. Included clearer buttons to simplify navigation between recordings, highlighting of audio segments as a place marker, and topic filters to make audio segments easier to find (Figure 4)
    User quotes
      “[The topic tags] need to be different colors.” [r2p02]
      “I didn’t even look – I looked up and saw the recordings [but not the account name they belonged to].” [r2p05]
- Round 3
  Updated prototype to include color-coded topic tags, new affordances for selected filters, ahead and back 10-second controls, more prominent play or pause button, clear filters button, and more prominent display of all account names the user has access to (Figure 5)
  User quotes
    “I was wondering how I could [turn the audio] off.” [r3p05]
    “Should you have some way to alert this [segment] is [about] the hand, the foot, blood pressure...” [r3p02]
- Round 4
  Updated prototype to make playback controls more prominent; added external links to MEDLINE Plus for medications, diagnosis, and test results topics; and added help link and popover dialog for filter controls (Figure 6)
  User quotes
    “Why is there so much? We have the same things [in the filters] as [in the list of audio segments]...Can we make it so [the filters are] clearly going to help [find audio segments]?” [r4p11]
    “Put a search in to specifically search for [the desired topic].” [r4p04]
- Round 5
  Updated prototype to move audio playback closer to top of the window, moved filters to a shopping-like sidebar, added text search or filter, added editable notes field to recordings, renamed the clear filters button, added a mute button, and added a button to go back to the beginning of recording (Figure 7)
  User quotes
    “It’s a little confusing whether [the text search] is part of [the topic filters].” [r5p03]
    “I did not see ‘Links to external information’ so I clicked on [the hyperlink].” [r5p04]
- Final user interface
  Improved understandability for playing a particular audio segment, clarified the distinction between clicking on a tag-row and clicking on an external information link, improved the usability of the text search and topic filter features, and reduced the number of information classes (Figure 8)

## Discussion

### Principal Findings

Following 5 rounds of user design sessions, we iteratively developed a highly usable interface that enables end users to seamlessly interact with desired functions, including playback and sharing of recordings, identifying key segments of the recording, and linking to trustworthy web-based resources. When presented with tasks to find specific topics in a visit recording, participants readily chose to use features (eg, tags, filters, and text search) that helped them more quickly find and play the audio related to that topic by simply listening to the entire recording. Participants were overwhelmingly positive about the concept of accessing a curated audio recording of a clinic visit; however, some participants reported concerns about privacy and the ability of participants to use a computer-based system to access recordings. Although patient partners felt that our final edits addressed confusion about the use of hyperlinks and playback in HealthPAL, further usability testing in less controlled settings is needed.

### Comparison With Previous Work

Previous studies of audio or video recording in the health care context have focused primarily on providing a hard copy of a recording to a patient, for example, a CD, cassette, or digital recorder [27]. In more recent developments, commercial apps have emerged that allow the recording, sharing, and tagging of audio recording; however, user-centered design of the app is rarely reported, and concerns about the collection and sharing of patient data have been raised [60].

An exception is *SecondEars*, a recording app developed by Lipson-Smith et al [61] for use by patients receiving cancer treatment. Similar desired features in a recording platform were identified using the MoSCoW (Must Have, Should Have, Could Have, and Won’t Have) method in their study. Interestingly, although the SecondEars app focuses on providing a simple recording of oncology clinic visits, patients noted that the ability to link notes to a particular section (ie, minute and second) of an audio recording would be desirable—a unique feature in HealthPAL. The HealthPAL design and our evaluation align with this previous work in that our iterative design incorporated many of these effective meeting browser features: a compressed view of the recording, showing key terms with segment boundaries, and text search of the transcript. Our methodology took learning effects into consideration in our focus on usability for first-time use of the system by including unique patients in each round. In addition, we increased the validity of our findings by asking participants to adopt roles that were reflective of real-world use, that is, participants playing the patient role listened to the recording before using HealthPAL, as patients would be part of the clinic visit in the real world.

It is likely that the inverted *U*-shaped distributions of performance were the result of a younger age demographic and use of paper prototyping in earlier rounds, in addition to the introduction of newer features in the low-fidelity prototype. Although participants became comfortable with most features, some features such as hyperlinks, filters, and the advanced search caused some confusion in the final round of testing. These challenges may be explained by a lack of familiarity with the modern UI design [62], especially in the absence of explicit feedback on actions. Previous usability studies have also reported that, although older adults understand hyperlinks, they can become disoriented when trying to use them [63], and it is unclear which elements of the display can be clicked. It is recommended that hyperlinks appear *touch* interactive [64]. In future iterations of HealthPAL, we will take these additional insights to further improve the usability of the system for older adults before evaluation.

Participants’ comments regarding the potential of HealthPAL to improve recall and understanding are supported by previous reviews, which found that sharing recordings can lead to such improvements [28]. However, previous research fails to determine the impact of sharing audio recordings on the ability of patients to manage their own care or the added value of annotated visit recordings. We plan to explore this knowledge gap through a pilot trial of our system.

### Limitations

Our sample was predominately White and college educated, reflecting the demographics of the region where our study took place. Further work is needed with individuals from more diverse ethnic and racial groups and from those with lower educational attainment levels. Our project was conducted in controlled settings, where participants were asked to think aloud and received assistance, if needed, with tasks. Although this is important at this stage of user design, it does not reflect the user experience in naturalistic settings. We plan to conduct further field testing in less controlled settings, where clinic visits will be recorded, annotated using our machine learning models, and used at home by patients. During this phase of testing, we will gather information on implementation factors and include clinician feedback. We will also obtain feedback from caregivers using actual clinic recordings of their loved ones’ visit. Some participants may have been unclear on the task instructions related to finding additional information (opening tabs outside of the UI), which may have resulted in the higher reported critical incidents in round 5 relative to our other usability metrics, that is, high SUS scores and task completion rate. In addition, our definition of *critical incident* was broad, including any change from the anticipated task path, not only those that resulted in task failure. Finally, we used a laptop computer for all usability testing sessions, but we hope to create a mobile adaptive UI, which will require further testing. Given the simplistic design concept, we believe that the interface can be quickly adapted to a mobile interface.

### Conclusions

Sharing visit recordings with patients is an emerging strategy for improving the transparency and communication of visit information. We have developed a highly usable audio PHL, HealthPAL, designed to allow patients and their caregivers to access easy-to-navigate recordings of clinic visits, with key concepts tagged and hyperlinks provided to further information. The interface has been rigorously co-designed with older adult patients and their caregivers and is now ready for further field testing. Our design work has identified and evaluated key features: a tag-based visualization for finer-grained playback of the visit recording coupled with tag-based filtering and text search on audio segments of the recording, which we believe will inform future design of such systems. The successful development and use of HealthPAL may help improve the ability of patients to manage their own care, especially older adult patients who have to navigate complex treatment plans.

## Abbreviations

AVS: after-visit summary
D-H: Dartmouth-Hitchcock
DHMC: Dartmouth-Hitchcock Medical Center
MoSCoW: Must Have, Should Have, Could Have, and Won’t Have
NLP: natural language processing
PHL: personal health library
SUS: System Usability Scale
TURF: Task, User, Representation, and Function
UI: user interface
